# Comparison of Two Porcine-Derived Materials for Repairing Abdominal Wall Defects in Rats

**DOI:** 10.1371/journal.pone.0020520

**Published:** 2011-05-26

**Authors:** Zhengni Liu, Rui Tang, Zhiyuan Zhou, Zhicheng Song, Huichun Wang, Yan Gu

**Affiliations:** Department of General Surgery, Shanghai Ninth Hospital affiliated to Shanghai JiaoTong University School of Medicine, and Hernia and Abdominal Wall Surgery Center of Shanghai JiaoTong University, Shanghai, China; Harvard Medical School, United States of America

## Abstract

**Objective:**

The purpose of this study was to compare the mechanical properties, host responses and incorporation of porcine small intestine submucosa (PSIS) and porcine acellular dermal matrix (PADM) in a rat model of abdominal wall defect repair.

**Materials and Methods:**

Prior to implantation, PSIS and PADM were prepared and evaluated in terms of structure and mechanical properties. Full-thickness abdominal wall defects were created in 50 Sprague-Dawley rats, and were repaired using either PSIS or PADM. Rats were sacrificed 1, 2, 4, 8 and 12 weeks post-repair and examined for herniation, infection, adhesions, contraction, and changes in the thickness and strength of the tissues incorporated at the defect sites. Histopathology and immunohistochemistry were performed to analyze inflammatory responses, collagen deposition and vascularization.

**Results:**

PADM showed more dense collagen deposition and stronger mechanical properties than PSIS prior to implantation (*P*<0.01). However, the mechanical properties observed after integration with the surrounding native tissues was similar for PADM and PSIS. Both PADM and PSIS showed significant contraction by week 12. However, PADM tissue induced less adhesion and increased in thickness more slowly, and showed less infiltration by foreign giant cells, polymorphonuclear cells, and mononuclear cells. Improved remodeling of host tissue was observed after PSIS implantation, which was apparent from the orientation of bands of fibrous connective tissue, intermixed with newly formed blood vessels by Week 12.

**Conclusion:**

PSIS showed weaker mechanical properties prior to implantation. However, after implantation PSIS induced more pronounced host responses and showed better incorporation into host tissues than PADM.

## Introduction

Currently, more than 70 types of mesh are available, and are classified as synthetic material or biological material according their particular composition [1]. Although permanent synthetic meshes can provide enough mechanical strength for use in abdominal repairs, their non-absorbable characteristics may cause potential problems resulting in infections, adhesions, erosion into the abdominal viscera, bowel fistulae, bowel obstruction and chronic pain, which can lead to more complex and costly surgery [2]. Biological meshes are acellular materials derived from humans or animals that have an intact extracellular matrix. The major advantages are that they allow host tissue invade and carry a lower risk of complications. Biological material from humans is of limited availability and carries a high commercial cost [3]. Xenogeneic materials, such as porcine-derived tissues, do not have such problems, therefore xenogeneic materials are now considered to be clinical useful for abdominal wall repair.

Porcine small intestine submucosa (PSIS) and porcine acellular dermal matrix (PADM) are two naturally-derived materials used for biological meshes that have been used clinically. PSIS is prepared from the submucosa of the small intestine of pigs in a manner that removes all cells, but retains the natural 3-dimensional (3D) composition of the extracellular matrix (ECM). The ECM acts as a scaffold into which cells can migrate and proliferate. PSIS has a high collagen (Types I, III, and V) content that forms the scaffolding for the ECM. The non-collagenous portion within PSIS contains numerous growth factors such as FGF-2, TGF-β and VEGF [4]–[5]. PADM prepared from porcine skin retains the intact structure of porcine dermal collagen, which is very similar in structure to human tissue. All non-collagenous material and cells are removed from the porcine tissues and only collagen and elastin, which retain the original 3D structure, remain [6]. The objectives of this study were to evaluate the morphological and mechanical properties of these two collagen-based materials *in vitro* and to compare their mechanical properties, host responses and tissue incorporation after implantation *in vivo*.

## Materials and Methods

### Experimental animals

Fifty male Sprague-Dawley rats, each weighing 200–250 g, were obtained from SLAC National Rodent Laboratory Animal Resources (Shanghai, China). All animal study protocols were approved by the Institutional review committee of Shanghai Jiao Tong University School of Medicine (ID: SYXK 2008-0050). Rats were housed in accordance with current national guidelines regarding animal welfare. The environment was maintained at 18–26°C with a relative humidity of 30–70%.

### Preparation of implants

#### PSIS

Fresh porcine small intestine was obtained from Fuxin abattoir (Shanghai, China). Segments of fresh porcine small intestine were cut into lengths of approximately 5 cm and flushed through with tap water to remove the intestinal contents. After longitudinal splitting of the intestinal segments, the serosal and muscular layers were removed mechanically and de-cellularized. This was done by shaking the small intestinal segments at 4°C in 1 L of 0.2% TritonX-100 (Sigma Chemical Co, St Louis, USA) containing 26.5 mmol/L ammonium hydroxide for 7 days. After de-cellularization, the small intestinal segments were washed in deionized water for 72 h. The resulting PSIS was freeze-dried at −55°C for 48 h and sterilized using γ-rays (30 kGy; ^60^Co, Shanghai Institute of Applied Physics, Chinese Academy of Sciences). The two-layer PSIS scaffolds were trimmed to a size of 50×40×0.2 mm^3^ prior to implantation. Samples were stored at −80°C until required.

#### PADM

Fresh porcine skin was obtained from Fuxin abattoir. After a thorough cleaning, excision of the sub-dermal fatty tissue and hair removal, the resulting derma was cut into pieces. The pieces were soaked in a solution containing 0.25% trypsin solution at 4°C for 24 h, followed by treatment with a 0.1% SDS solution at room temperature for 6 h, and further treatment with 560 units/L of Dispase solution at 4°C for 24 h. Finally, the tissue was treated with 0.1% SDS solution at room temperature for 6 h before being washed twice in PBS buffer for 30 min. The porcine derma was then cross-linked using glutaraldehyde to prevent rapid degradation and 50×40×0.5 mm^3^ sections were uniformly punched to promote the ingrowth of abdominal wall tissue after implantation. Samples were preserved in PBS buffer after sterilization with γ-rays (30 kGy).

### Scanning electron microscopy (SEM) examination of the scaffolds

Samples were freeze-dried, loaded onto aluminum studs and coated with a thin layer of gold for 3 min at 8 mA at 0.1 Torr. The collagen morphology was then examined under a scanning electron microscope (Ulitra55, Zeiss, Germany). Samples were scanned and the micrographs recorded. The morphological arrangement of the collagen fibers in the two materials after acellular treatment was compared.

### Surgical procedure

Each Sprague-Dawley rat was anesthetized using an intramuscular injection of ketamine (60 mg/kg). The abdominal skin was shaved and disinfected with a povidone iodine solution. A midline incision, 50 mm in length, was performed along the linea alba and a 30×20 mm^2^ full-thickness defect, including the fascia, muscles and peritoneum, was created centered on the midline. The rats were then randomly assigned into PSIS and PADM groups (n = 25) and a 50×40 mm^2^ section of sterile PSIS or PADM was implanted into each rat to repair the abdominal wall defect. Each side of the scaffold overlapped the edge of the defect by 10 mm and was fixed using 3-0 polypropylene interrupted sutures. The skin was then closed using 2-0 Vicryl interrupted sutures. No antibiotic treatment was given during the experiments.

### Macroscopic examination

Five rats from each group were randomly sacrificed 1, 2, 4, 8 and 12 weeks after implantation. Any evidence of seroma, hematoma, or infection of the implants on the subcutaneous and visceral sides was noted upon gross examination before sampling. The implant dimensions were measured using a centimeter scale and recorded to assess any subsequent intra-corporal contraction. The abdominal wall was distended with 200 ml saline and any abnormal abdominal protrusion, before or after mechanical examination, was considered to be a fascial weakness or a hernia [7]. The tenacity of any adhesions and the percentage of the implants covered by adhesions were scored as described by Jenkins [8] (Table 1). After sampling, the thickness of the implant was measured by taking five random measurements over the central part of the implant and changes in thickness (Δ) were defined as: themean value measured at the time of sacrifice – the original thickness of the implant. All specimens were evaluated in a blinded fashion by the same individual to ensure consistency.

**Table 1:** Semiquantitative adhesion scoring system.

Score	Tenacity	Surface area
0	No adhesions	No adhesions
1	Minimal adhesions freed by blunt	<25%
2	Moderate adhesion freed by aggressive	<50%
3	Dense adhesion freed by sharp	<75%
4	-	≥75%

### Histological examination

Serial sections of the explants (the implant plus 1 cm of the surrounding tissues and the interface) were embedded in paraffin, cut into 3 µm sections and stained with hematoxylin and eosin. Microscopic evaluation was performed to quantify the number of foreign body giant cells, polymorphonuclear cells (PMNs) and mononuclear cells (MNs) invading the implanted scaffolds. Qualitative assessment of collagen deposition was performed using the Masson trichrome stain. The amount of inflammatory cell infiltration and the amount and organization of any collagen deposits were scored using a method analogous to that described by Badylak [9] (Table 2). Five fields per section were counted by the same individual at ×400 magnification (E600, Nikon, Japan) in a blinded fashion.

**Table 2:** Histologic scoring criteria for microscopic examination.

	Score			
Category	0	1	2	3
Foreign body giant cells	0	1–5	6–10	>10
Polymorphonuclear cells	0	1–5	6–10	>10
Mononuclear cells	0	1–5	6–10	>10
Collagen				
Organization	Totally disorganized	Slightly organized	Moderately organized	Well organized
Amount	0	Mild	Moderate	Abundant
Vascularity	0	1–3	4–10	>10

Immunostaining was performed to assess any neovascularization of the implants. Samples were incubated with an anti-CD31 antibody (1∶200; Abcam, Cambridge, MA) for 60 min. Sections incubated without the primary antibody served as negative controls. The mean percentage area of blood vessels (% A_bv_) was calculated for 10 randomly selected high-contrast fields at ×200 magnification using Image-Pro Plus (v. 6.0) as follows: percentage area of blood vessels = area of capillary vessels/total tissue area. All evaluations were performed by one pathologist who was blinded to the materials being tested.

### Examination of mechanical properties

#### Mechanical properties before implantation

Mechanical examination of the 50×10 mm^2^ samples was conducted at room temperature using a uniaxial materials testing machine (Instron Model 5542, Canton, USA). The length of the tested sample, held between two grippers, was set at 25 mm. Samples were stretched along their longitudinal axes at a cross-head speed of 10 mm/min until failure. The load and displacement were recorded throughout the elongation and converted to a stress-strain curve based on the initial specimen dimensions. The stiffness (N/mm) was determined by calculating the slope of the load (linear portion) vs. displacement. Five samples of each material were tested.

The bursting strength of the materials was examined using a bursting strength tester (Mullen tester, USA) according to ASTM Standard [10]. Examinations were conducted using specimens conditioned in a standard atmosphere for testing materials. The conditioned specimen was inserted under a tripod, drawn taut across the plate, and clamped in place. The liquid pressure (psi) was then increased at a uniform rate of 90 ml/min until the specimen ruptured. This test method measured the resistance of the materials to bursting.

#### Mechanical properties after implantation

The load on the explants was measured as describe above. All of the explants tore at the interface during tensiometry testing. The reported values do not actually represent the strength of the material, but rather represent the degree of integration at the interface with the surrounding tissues. This is known as the “strength of incorporation” [11]–[12].

### Statistical analysis

All data were expressed as the mean ± SD. The difference between PSIS and PADM in terms of mechanical properties, gross evaluation and host response were analyzed using the Student's t-test. The statistics package for social science (SPSS version 13.0; SPSS Inc., Chicago, IL, USA) software was used for statistical analysis. *P*<0.05 was determined to be statistically significant.

## Results

### Scaffold preparation and SEM measurements

**Figure 1:**
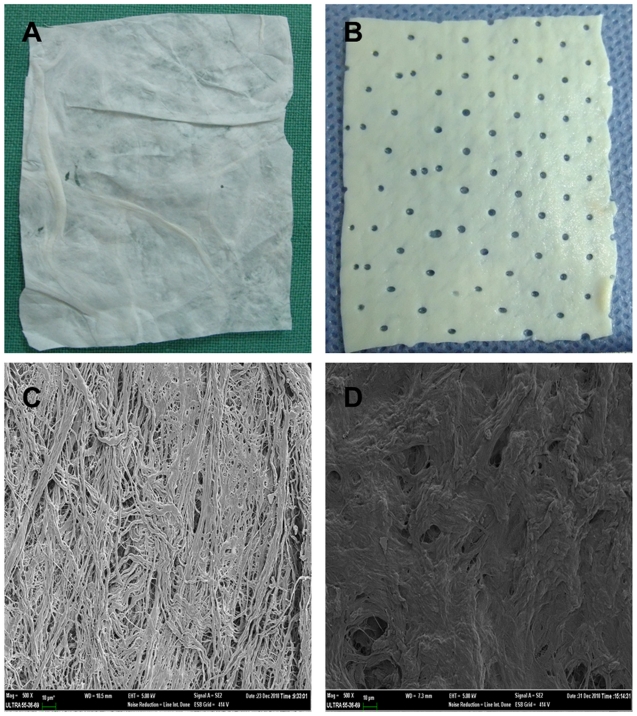
Scaffolds structures by SEM. A) PSIS scaffold. B) PADM scaffold. C) SEM photographs showing PSIS, ×500 and D) PADM, ×500.

PSIS and PADM were successfully prepared as shown in Fig. 1A and B. SEM measurements showed that the collagen fibers within PSIS formed a loose meshwork (Fig. 1C), while those within PADM were more dense (Fig. 1D).

### Mechanical properties prior to implantation

PSIS and PADM were tested for maximal loading, maximal displacement, stiffness and bursting strength (Table 3). The mean maximal load, stiffness and bursting strength of PADM were significantly higher than those for PSIS (*P*<0.01). However, PSIS had better extension properties than PADM (*P*<0.01).

**Table 3:** Mechanical properties of PSIS and PADM.

	Maximum Load(N)	Maximum Displacement(mm)	Stiffness(N/mm)	Bursting Strength(Psi)
PSIS	22.81±2.54**	6.88±2.59**	9.12±1.45**	23.00±1.15**
PADM	43.16±2.53	2.47±0.78	22.41±4.09	56.67±4.16

**vs **PADM(** ***P*** **<0.01).**

**Psi = 6.895 Kpa.**

### Clinical evaluation of experimental animals

All rats recovered normally from surgery and survived to their predetermined sacrifice date. None of the rats showed any evidence of bulging or herniation at the implantation site either before or after instillation of 200 ml saline. Seroma were noted in two cases in the PSIS group and four cases in the PADM group 2 weeks after implantation. Intestinal obstruction was noted in one case in the PADM group 2 weeks after implantation. Further gross examination showed evidence of a small intestinal obstruction secondary to an adhesion, resulting in a kink in the intestine at the site of the central portion of the implant. There were no clinical signs of hematoma or infection in either group.

### Macroscopic observation after implantation

**Figure 2:**
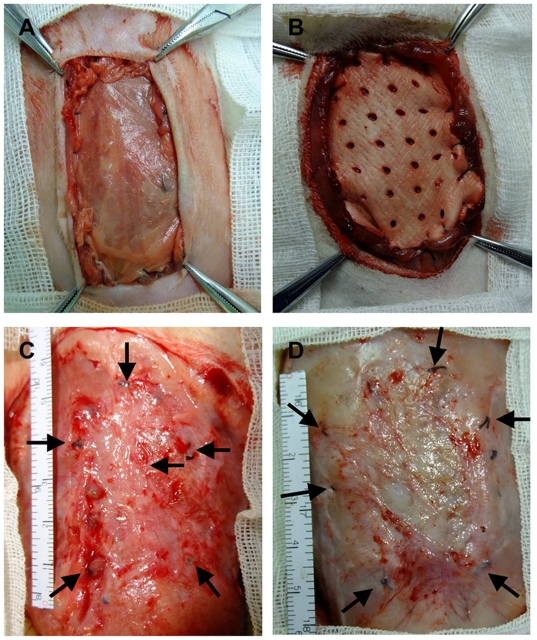
Contraction of the implants 12 weeks after implantation. The contraction of the implants surrounding the sutures (arrows) repairing the abdominal wall defects. A) PSIS implant. B) PADM implant. C) Contracted PSIS implant 12 weeks after implantation. D) Contracted PADM implant 12 weeks after implantation.

The surface area of the implants was significantly lower in both the PSIS (14.2±2.3 cm^2^) and PADM (12.6±3.1 cm^2^) groups 12 weeks after implantation than it was prior to implantation (20 cm^2^) (*P*<0.01). The contraction rate was 29% for PSIS and 37% for PADM. There was no significant difference in surface area contraction between the PSIS and PADM groups after implantation (*P*>0.05; Fig. 2 and Table 4).

**Table 4:** Surface area of PSIS and PADM.

Materials	Pre-implant area(cm^2^)	Mean post-implant area(cm^2^)	Mean contraction (%)	P value
PSIS	20	14.2±2.3	29±12	<0.01
PADM	20	12.6±3.1	37±16	<0.01

Both PSIS and PADM showed significant contraction after implantation.

**Figure 3:**
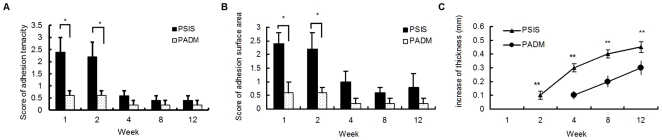
Adhesion and thickness of scaffolds varied during 12 weeks. A and B) Adhesion tenacity and surface area of PSIS and PADM after implantation. C) The increase of the implant thickness after implantation.*P<0.05 *vs.* PADM. **P<0.01 *vs.* PADM.

Adhesion between the implants and the peritoneal contents was significantly higher in the PSIS group than in the PADM group during the first 2 weeks (*P*<0.05), and the tenacity of the adhesions and surface area scores in the PSIS group were almost 4-fold those in the PADM group. Two weeks after implantation, the level of adhesions in the PSIS group decreased dramatically, and was not significantly different from that in the PADM group. Meanwhile, the adhesions in the PADM group were mild, and decreased slightly between 2 to 12 weeks after implantation (Fig. 3A and B).

The thickness of the implants 12 weeks after implantation was significantly greater in both PSIS (0.65±0.04 mm) and PADM (0.8±0.05 mm) groups than it was prior to implantation (PSIS, 0.2 mm; PADM, 0.5 mm; *P*<0.01). The increase was significantly higher in the PSIS group than that in the PADM group between 2 and 12 weeks after implantation (*P*<0.01, Fig. 3C).

### Histological observations after implantation

**Figure 4:**
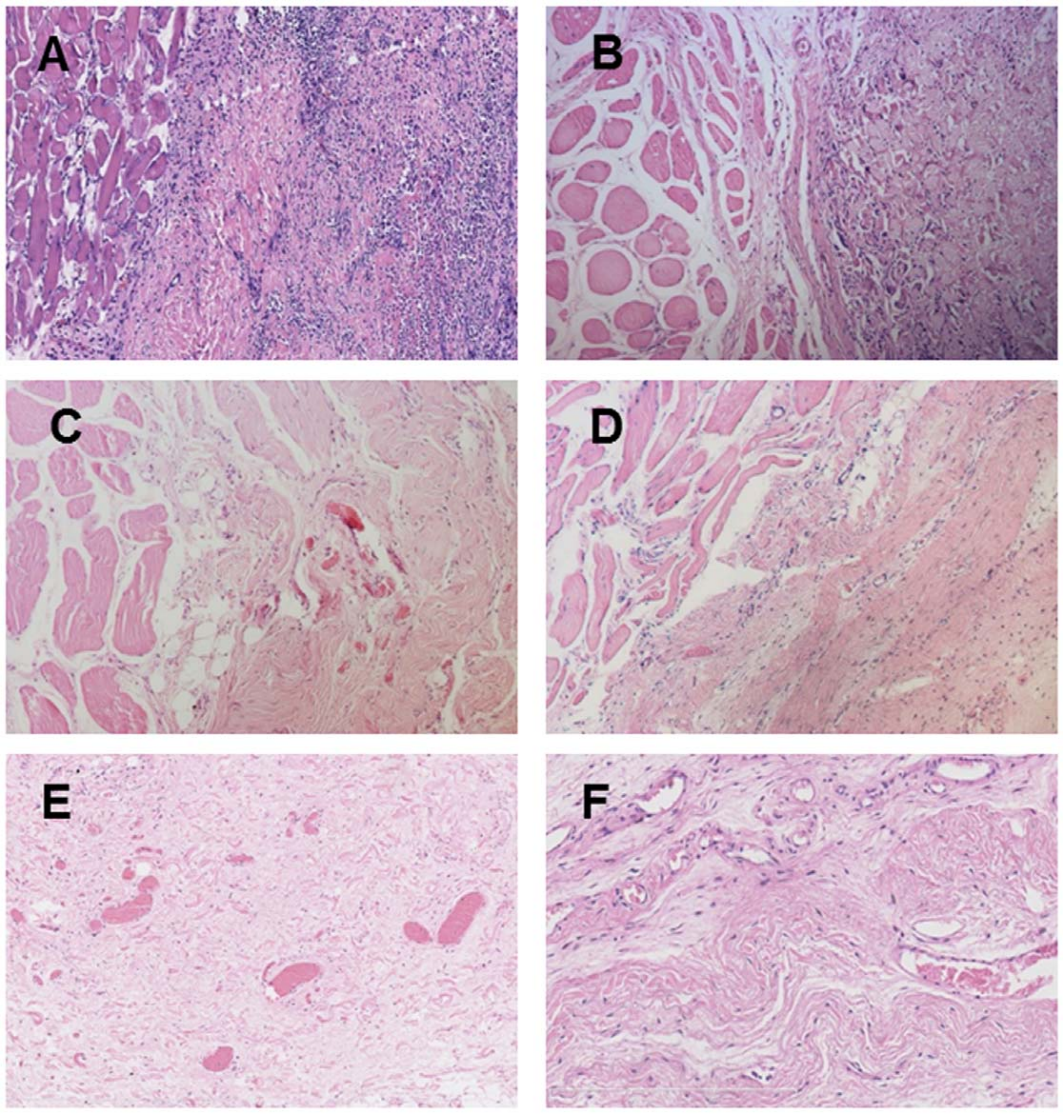
Histologic appearance of the PSIS and PADM explants (H & E staining). (A) PSIS at Week 1 (×100). Pronounced inflammatory cell infiltration and few newly formed blood vessels were observed at the interface between PSIS and the surrounding tissues. (B) PADM at Week 1 (×100). Less inflammatory cell infiltration and fewer newly formed blood vessels were observed at the interface between PADM and the surrounding tissues. (C) PSIS at Week 12 (×100). The inflammatory response diminished significantly, and a large amount of well-vascularized, fibrous connective tissue, was observed at the interface. (D) PADM at Week 12 (×100). A similar level of inflammatory response was observed, but with less vascularization at the interface compared with PSIS. Host incorporation (invasion of the implant by host fibroblasts and endothelial cells) was also observed. (E) PSIS at Week 12 (×200). Oriented bundles of collagenous connective tissue with abundant newly formed blood vessels were observed, with only a little PSIS remaining at the center of the scaffold site. (F) PADM at Week 12 (×200). Oriented bundles of collagenous connective tissue with some newly formed blood vessels were observed, with more PADM remaining at the center of the scaffold site.

**Figure 5:**
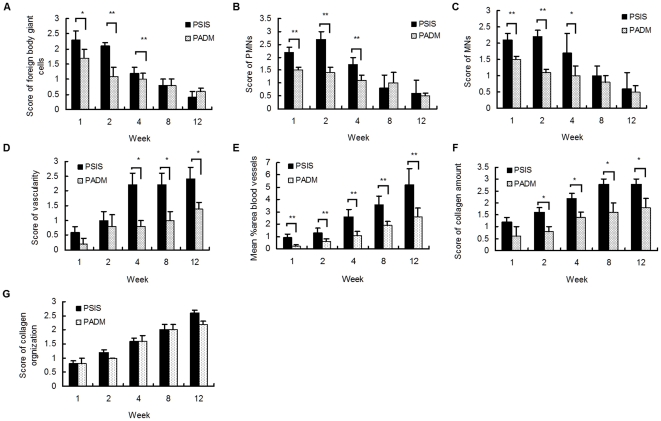
Histological analysis of PSIS and PADM 1 to 12 weeks after implantation. A) Statistical analysis of the scores for the number of foreign body giant cells, B) PMNs, C) MNs, D) the amount of vascularization, E) the blood vessels density, F) the amount of collagen, and G) the collagen organization within the implants. * *P*<0.05 *vs.* PADM. ** *P*<0.01 *vs.* PADM.

Microscopic analysis showed that infiltrating inflammatory cells (foreign body giant cells, PMNs and MNs) appeared 1 week after implantation. Rats in the PSIS group showed a pronounced inflammatory response 1 to 4 weeks after implantation, which was significantly greater than that seen in the PADM rats (*P*<0.05). These inflammatory reactions gradually became weaker and fell to negligible levels (similar to those in PADM rats) by 12 weeks after implantation (*P*>0.05). Overall, the PADM rats showed substantially lower levels of inflammatory cell infiltration over the 12 weeks (Fig. 4 and Fig. 5 A–C).

**Figure 6:**
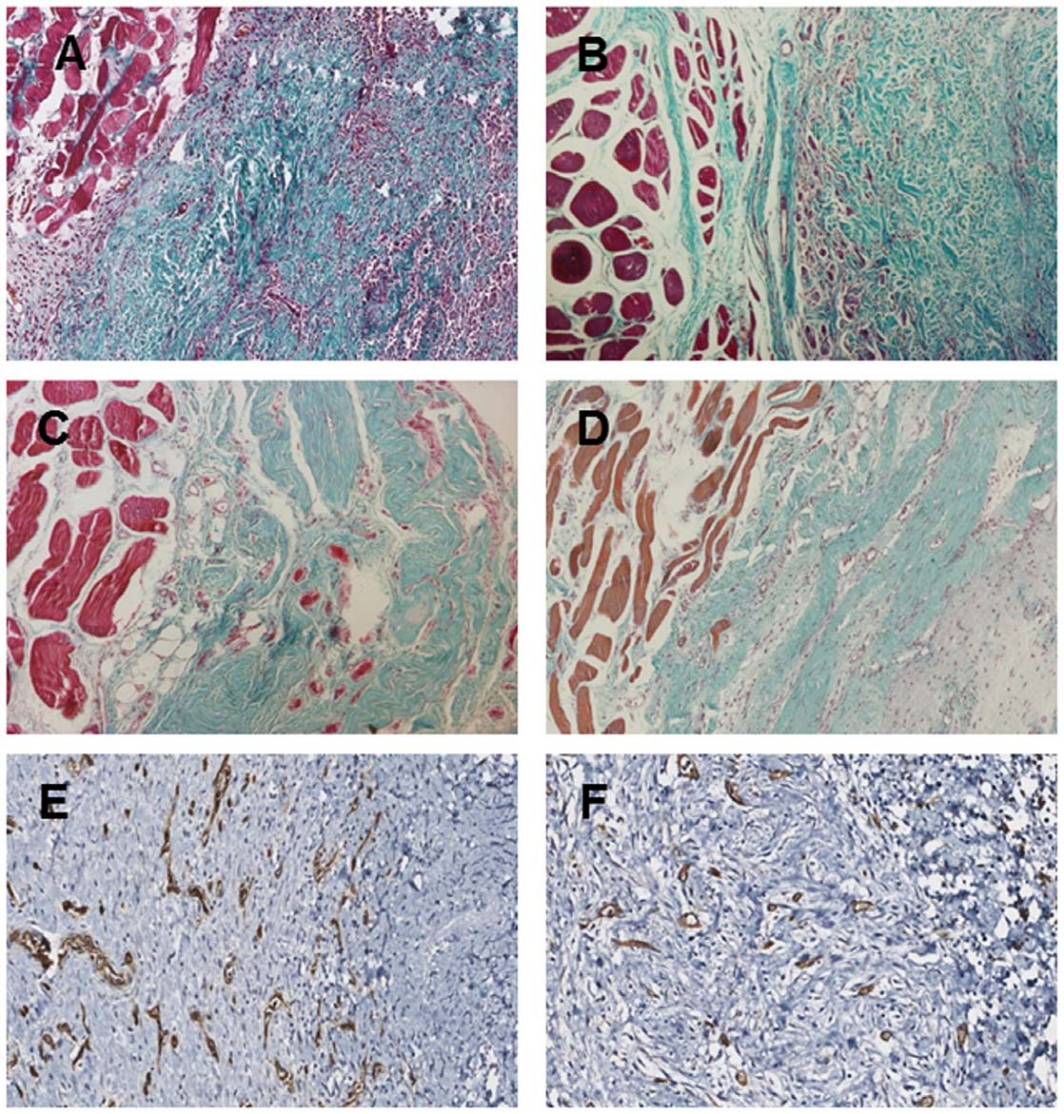
Histologic appearance of the PSIS and PADM explants (Masson trichrome staining and Immunostaining). A–D: Masson trichrome staining; E–F: Immunostaining. (A) PSIS at Week 1 (×100). Only a thin layer of disorganized collagen deposition was observed at the interface. (B) PADM at Week 1 (×100). Thinner, but more organized, collagen deposition was observed at the interface. (C) PSIS at Week 12 (×100). At the interface, the implants were significantly degraded and replaced by a thick layer of well-vascularized and organized fibrous connective tissue. (D) PADM at Week12 (×100). At the interface, the implants were replaced by well-organized collagen deposition similar to that seen for PSIS. (E) PSIS at Week 12 (×200). Active neovascularization was observed in the center of the scaffold. (L) PADM at Week 12 (×200). Fewer new blood vessels grew into the center of the PADM scaffold compared with the PSIS implants.

The implanted tissues were incorporated into the host tissues of both groups, as defined by the in-growth of new blood vessels and the amount of new collagen deposited within the implants [13] (Fig. 4E–F). Few new blood vessels were noted in either group within the first 2 weeks; however, PSIS rats showed significantly increased levels of neovascularization from 4 to 12 weeks (*P*<0.05). Lower levels of blood vessel ingrowth were observed in PADM rats up until Week 12. The density of capillary ingrowth, as indicated by %A_bv_, was significantly higher in the PSIS group (5.2±1.3%) than that in the PADM (2.6±0.7%) group during the entire experimental period (*P*<0.01**;** Fig. 5D–E and Fig. 6 E–F).

Masson trichrome staining showed that PSIS induced more intense collagen deposition from 2 to 12 weeks than PADM (*P*<0.05). There was no significant difference in collagen organization between the PSIS and PADM groups after implantation (*P*>0.05; Fig. 5F–G and Fig. 6A–D).

### Mechanical properties after implantation

**Figure 7:**
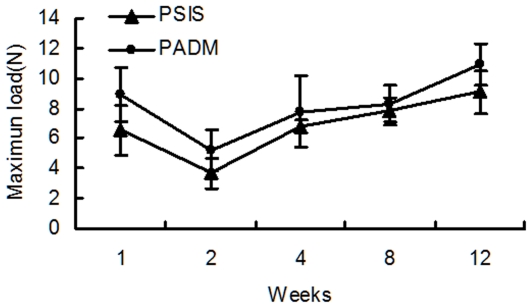
The strength of incorporation of the PSIS and PADM implants during the course of the experiment.

The strength of incorporation of the PSIS and PADM explants decreased in the first 2 weeks, and then increased gradually over the following 10 weeks. There was no significant difference between the maximal loads borne by the PSIS and PADM explants (*P>*0.05; Fig. 7).

## Discussion

Several xenogeneic biological materials, including bovine dermis, bovine pericardium, ovine dermis, PSIS and PADM, are available for the tension-free closure of abdominal wall defects in cases where a synthetic mesh is not indicated or where inadequate amounts of autogenous musculofascial tissue are present [9]. PSIS and PADM are two of the most commonly used porcine-derived biological materials [12]. The present study provides both quantitative and qualitative information regarding the morphological and mechanical properties and host tissue responses after the implantation of PSIS and PADM.

The implantation of PSIS, an acelluar collagen-based biodegradable matrix derived from the submucosal layer of the porcine intestine, resulted in minimal immune responses in rats. PADM, isolated living cells and non-collagenous material, represents intact porcine dermal collagen and retains the original 3D structure of the dermis. Each of these materials is characterized by its own distinctive physical, mechanical, and biological properties. Our results demonstrated that both PSIS and PADM maintained sufficient strength and incorporate host tissues to repair abdominal wall defects efficiently. There was no evidence of bulging or herniation at the defect sites in either group during the post-operative observation period, even after instillation of 200 ml saline into the peritoneal cavity. The test of infiltrating saline was carried out to evaluate the properties of implants that response to the burst increasing of intra-abdominal wall pressure. The results revealed that the increase in intra-abdominal pressure still did not exceed abdominal wall counterpressure after implantation, though testing the entire implants using bursting strength tester *in vivo* was the optimal method to evaluate the mechanical properties for two groups which was limited by operation in this study. An increase in the thickness of the implants appears to be necessary to maintain body abdominal wall integrity during remodeling and biological degradation of the scaffold. Our results showed that both PSIS and PADM were gradually replaced by connective tissue; developing a thicker abdominal wall layer by the end of the observation period. This increase in implant thickness was particularly evident for PSIS, which is consistent with the greater inflammatory responses observed with this implant. Contraction of the implants may reflect an inadequate rate of vascularization, leading to inadequate nutrition and subsequent necrosis and fibrosis [13]. In our study, both the PSIS and PADM implants had a significant contraction during 12 weeks after implantation. This suggests that insufficient neovascularization during the early stages post-implantation may lead to the contraction of the implants. Implant adhesion is another important criterion for the reconstruction of the abdominal wall, and is mainly caused by bleeding and inflammation during the repair process. One factor leading to the formation of adhesions is the inflammatory response induced by the implanted materials. During inflammation, plasminogen activator is suppressed and deposition of a fibrin matrix is increased, which gradually turns into organized fibrous adhesions [14]. Our results showed that the adhesion associated with PSIS were more extensive and severe than those associated with PADM, which was also in accordance with the increased inflammatory response associated with PSIS observed at the repair site.

Further histological evaluation showed that the host responses and incorporation of PSIS were different from those observed from PADM. PSIS implants caused a more pronounced inflammatory response, as evidenced by infiltration by both PMNs and MNs during the initial stages; however, this inflammatory response rapidly diminished to a level similar to that seen in PADM implants, which induced a less severe inflammatory response throughout of study period. Compared with the PADM implants, the PSIS implants degraded more quickly and were almost totally replaced by organized collagenous tissues. Little PSIS material remained after 12 weeks, which suggests a better remodeling process. This was supported by the finding of prominent, well-vascularized and organized fibrous connective tissues with PSIS, in contrast to the lack of well-vascularized fibrous connective tissue orientation seen with PADM. The original PSIS scaffold material was no longer evident. Instead, well-organized, oriented bands of fibrous connective tissue were present.

These results demonstrate that the use of PSIS results in better host incorporation and remodeling in terms of the amount and organization of the deposited collagen, improved neovascularization, a looser 3D meshwork and growth factors contained, all of which may promote host tissue repairing and remodeling compared with PADM. These results are consistent with those of Mattia et al, which focused on commercial PSIS and PADM [12].

The strength of incorporation is a well-established measurement of the incorporation of host tissues into an implant [12]. Our results showed that the PSIS scaffold was significantly weaker and less stiff than that of PADM; however, *in vivo* studies showed that the strength of PSIS was similar to that of PADM, and there was no significant difference in the maximal loads borne by PSIS and PADM after implantation. PSIS possesses a “looser” structure than PADM which has a thicker and denser collagen arrangement. However, although the cross-linking is believed to stabilize the implant by preventing degradation by collagenases, they may also change a potentially biologically interactive material into a relatively inert material, preventing repopulation and remodeling by host cells [15] and resulted in limited host incorporation and, eventually, to decreased strength of incorporation.

In summary, our data suggest that both PSIS and PADM were well tolerated for a period of 12 weeks following implantation into Sprague-Dawley rats. PSIS was superior to PADM with respect to implant thickness, host incorporation including vascularization and collagen deposition, which all correlated well with the materials physical properties. Although the mechanical properties of the PADM scaffold were superior to those of PSIS, there was no significance difference in the strength of incorporation between these two materials *in vivo*. Our study shows that PSIS is a viable alternative to existing scaffold materials for the repair of abdominal wall defects, and elicits a distinctive host tissue response. Apparently, the 2-layer PSIS we prepared may possess insufficient mechanical strength needed for the clinical study. The commercial biomaterial of PSIS are much thicker to provide adequate strength for the repair of abdominal wall defects owing to its multi-layered and cross-linked structure at the costs of moderate host incorporation *in vivo*. Further long-term studies are needed to determine the mechanical changes, incorporation into adjacent soft tissues and techniques to reduce the development of adhesions. The balance will be maintained by using various multi-layered and non-cross-linked PSIS. Furthermore, the application of tissue engineering technology to PSIS may be helpful in the development an ideal abdominal wall defects repair material.
